# Infected Hydatid Cysts Bacteria in Slaughtered Livestock and Their Effects on Protoscoleces Degeneration

**DOI:** 10.5812/jjm.10135

**Published:** 2014-06-01

**Authors:** Mohammad Fallah, Abdollah Kavand, Rasoul Yousefi Mashouf

**Affiliations:** 1 Department of Parasitology and Mycology, Hamedan University of Medical Sciences, Hamedan, IR Iran; 2 Kowsar Hospital, Social Security Organization, Borujerd, IR Iran; 3 Department of Microbiology, Hamedan University of Medical Sciences, Hamedan, IR Iran

**Keywords:** Hydatid cyst, Animals, Bacterial Infection

## Abstract

**Background:**

The protoscoleces of fertile hydatid cysts are considered as major risks in surgery and producing secondary cysts if rupture the cyst during operation and, cause infecting the dogs with adult worm if eaten by this animal. Bacterial infection of the hydatid fluid can lead to sterilization of the cyst.

**Objectives:**

The aim of this study was to determine the bacterial infection rate of livestock hydatid cysts in Hamedan, Iran, and test the isolated bacteria effects on viable protoscoleces, *in vitro*.

**Materials and Methods:**

A total of 5709 slaughtered livestock were inspected to detect the presence of hydatid cysts. The hydatid fluid of all cysts was cultured separately to isolate and identify the bacteria. The effect of isolated bacteria was tested on viable protoscoleces in culture tubes, *in vitro*. The culture tubes were observed and examined under a light microscope every two hours for 24 hours, and then, after 36 and 48 hours.

**Results:**

Infected cysts were found in 74% of animals in Hamedan (46% were calcified and the bacteria was isolated from 52%) and 62% in Borujerd. The isolated bacteria in the infected cysts were as follows: *Escherichia coli*, *E. blattae*, *Klebsiella pnoumoniae*, *Proteus mirabilis*, *Enterobacter aerogenes*, coagulase-positive and coagulase-negative Staphylococci, *Pseudomonas aeruginosa* and *Edwardsiella tarda*. The protoscoleces incubated with the isolated bacteria totally degenerated, but 55% of the protoscoleces in the control groups were intact and viable even after one week.

**Conclusions:**

This study indicated a high percentage of cysts bacterial infections in two provinces of Iran. The common isolated bacteria were *E. coli* and *Klebsiella*. The isolated bacteria degenerated the protoscoleces during short-time incubation, *in vitro*.

## 1. Background

Hydatid cyst or hydatidosis is an infection caused by *Echinococcus granulosus*. Typically, mature hydatid cyst produces numerous protoscoleces, each having the potential to develop into an adult worm after being ingested by a suitable definitive host, such as dog (1-3). Hydatid cyst is typically filled with a clear fluid (hydatid fluid) which is bacteriologically sterile (4). Outside-released protoscoleces have the ability to differentiate into hydatid cysts in viscera, and cystic differentiation of protoscoleces can probably be triggered by altered physiological conditions, such as bacterial diffusion into the cyst fluid (5). There is also direct evidence from cysts in livestock that protoscoleces show cystic development within the cyst (daughter cyst), degenerated by bacterial infection (6).

The fluid within the primary cyst is frequently more dense and dark, indicating the presence of some sort of debris or infiltrate. Some workers referred to this material as “matrix”, and at surgery, such cysts are frequently full of pus or leukocyte infiltrate and debris from a degenerated primary cyst (4). Bacterial infection is also sometimes present, leading to sterilization of the cyst (no protoscoleces production). Clearly, if pus exists, cellular infiltrate or bacterial cells will be present in the cyst cavity, and the germinal layer and cyst wall may not be intact. If a cyst ruptures, the sudden release of its contents may precipitate allergic reactions ranging from mild to fatal anaphylaxis, bacterial infection may occur, and there will be spread of protoscoleces, which may result in a multiple secondary hydatidosis (2, 7). There are few studies on the bacterial infections of hydatid cysts in animals and human, as well as the type of bacteria (8-11). Despite the degenerating effects of bacteria enter and reproduce in the hydatid fluid, there is no study on these effects, *in vitro*. The degenerative effects are due to direct invasion of the bacteria to the protoscolox body, or the exotoxin or enzymes secreted by the parasite, which are not clear yet.

## 2. Objectives

The aim of this study was to determine the bacterial infection rate of livestock hydatid cysts in Hamedan and Borujerd, and identify the type of bacteria. Moreover, test the isolated bacteria degenerating effects on viable protoscoleces, *in vitro*.

## 3. Materials and Methods

### 3.1. Geographic Status of the Area

The areas of this study were Hamedan and Borujerd cities, located in northern and southern slopes of Zagrus Mountains, Iran, a major sheep rising area with Mediterranean climate the average altitude of these areas is about 1330 m.

### 3.2. Parasitology

A total of 5709 livestock (481 cattle, 2127 sheep and 251 goats in Hamedan, and 553 cattle, 1944 sheep and 353 goats in Borujerd) from daily slaughters were inspected for presence of hydatid cysts, in Hamedan and Borujerd slaughterhouses. Lungs and livers of each animal were collected at the slaughter, individually identified, and maintained, until the cyst analysis was performed within the next three hours. Cysts of each organ were counted and measured. All cysts larger than 1 cm in parenchyma of the lungs and the livers were examined. The location, cyst number, diameter, fertility (fertile or infertile based on protoscolex presence), and bacterial infection status were recorded. Normal cysts were full of clear fluid, had a distinct white membrane, and one or more separate chambers. However, newly infected cysts were full of turbid fluid, and sometimes they were caseified or calcified.

The total cyst fluid was extracted from each cyst using a disposable syringe, and the volume was determined. Gravity sediment materials were incubated in a test tube in an incubator (37˚C) for a minimum of 30 minutes. The sediment from each cyst fluid was observed under a light microscope for protoscoleces, which were then tested for viability. Viability of the protoscoleces was assessed by body contractions, motility of flame cells, ease of staining with 0.1% aqueous eosin solution, and examination under a light microscope. Died protoscoleces took the dye, whereas the live ones did not (12). From each cyst, 1 mL of the cyst fluid containing protoscoleces was examined, and the protoscoleces were characterized as live (clear) or dead (red).

### 3.3. Bacteriology

The hydatid fluids of all collected cysts were separately cultured for isolation and identification of bacteria at the genus level; however, some bacteria were identified at the species level. The organisms were cultivated and isolated according to the methods outlined in Bailey & Scott’s Diagnostic Microbiology (13). Briefly, the hydatid cyst surface was washed by sterile normal saline and cleaned by ethanol. The hydatid fluid was aspirated by a syringe, and inoculated on blood agar (Merck, Germany) for detection of aerobic and facultative anaerobic Gram-positive bacteria, eosin methylene blue (EMB) agar and Makonkey agar for Gram-negative bacteria, and thioglycullate broth for anaerobes. 

The cultures were incubated in 37˚C for 24-48 hours, and then the colonies were removed for further studying. After 48 hours, smears were prepared from each colony and stained for microscopic examination. Biochemical and antigenic properties of the isolates were verified for identification purposes (14). The biochemical tests used for identification of the Gram-positive bacteria were: catalase, oxidase, DNase, coagulase and phosphatase reaction, mannitol fermentation, aesculin hydrolysis, starch and sodium hippurate, pyrrolidonyl arylamidase (PYR) and Christie-Atkins, Munch-Petersen (CAMP) tests, and novobiocin sensitivity. 

The biochemical tests used for identification of Gram-negative bacteria were: motility, urease, Voges-Proskauer, methyl red, indole production, potassium cyanide (KCN), o-nitrophenyl-β-d-galactopyranoside (ONPG) and H_2_S production, triple sugar iron agar (TSI), lactose fermentation, phenylalanine reaction, and lysine and ornithine decarboxylase tests. In essential cases, a specific antiserum against the bacteria was used for precise identification of the bacterial type (14). Finally, the isolates were cultured on differential media and the bacteria were identified at the genus or species level, whenever possible.

### 3.4. Protoscoleces and Isolated Bacteria

Effects of the isolated and cultured bacteria were tested on the protoscoleces in culture tubes, *in vitro*. A total of 1000 viable protoscoleces were added to a culture tube containing soy broth trypticase and the isolated bacteria, and other tubes empty of bacteria were prepared as controls. All the tubes were incubated at 37˚C. The culture tubes were observed and scanned microscopically every two hours for 24 hours, and then, after 36 and 48 hours. The analysis unit was the individual animal; the cysts statuses were also analyzed depended on the cysts origins (type of animal). For all the analyses, cysts containing protoscoleces were defined as fertile, and the cyst fluid containing bacterium was defined as “infected”. The fertility calculation was performed using only fertile cysts as the denominator. SPSS software version 15, IBM Chicago was applied to analyze the data.

## 4. Results

In general, hydatid cysts were found in 6.5% of animals from Hamedan and 7% from Borujerd. Localizations of cysts were 42.34% in lung, 46.93% in liver, and 10.7% in both liver and lung. In the Hamedan and Borujerd slaughterhouse, only 20% and 32% of the cysts were fertile, respectively. Fertilities of cysts in different animals were: 21.4% in sheep, 16.6% in cattle, and 0% in goats in Hamedan, and 35.75% in sheep, 22.64% in cattle, and 0% in goats in Borujerd. Infected cysts were found in 74% of animals in Hamedan (46% were calcified and the bacterium was isolated from 52% of cysts). Infected cysts rates were 88% in cattle, 70.7% in sheep, and 33.3% in goats. Infected cysts were found in 62% of animals in Borujerd, and bacterial infection rates were 70% in sheep, 43.4% in cattle, and 33.3% in goats. 

Isolated bacteria in the infected cysts of Hamedan animals were as follows: *Escherichia coli*, *E. blattae*, *Klebsiella pneumonia*, *Proteus mirabilis*, *Enterobacter aerogenes*, coagulase-positive and coagulase-negative Staphylococci, *Pseudomonas aeroginosa* and *Edwardsiella tarda*. The most common bacteria were *E. coli* (23.94%) and *K. pneumonia* (22.5%) in Hamedan, and *E. coli* (35.7%) and *K. pneumonia* (42.8%) in Borujerd. Regarding the organs, the most common isolated bacteria in liver were *E. coli* and *Klebsiella* in Borujerd and coagulase-negative Staphylococci in Hamedan. In Hamedan 63% of the isolated bacteria were toxinogenic; however, in Borujerd this rate was 80%.

**Figure 1.**
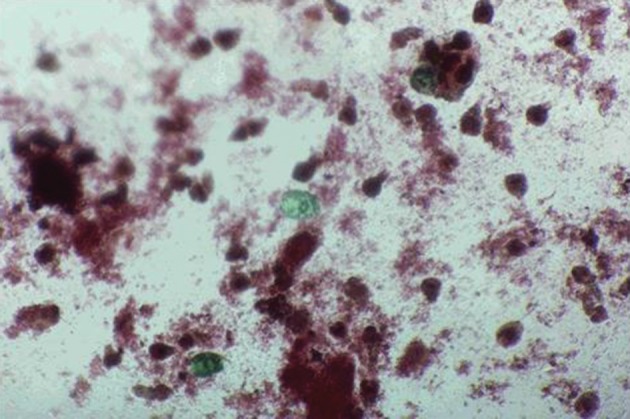
Protoscoleces After 12 h Exposure to Isolated Bacteria

**Figure 2.**
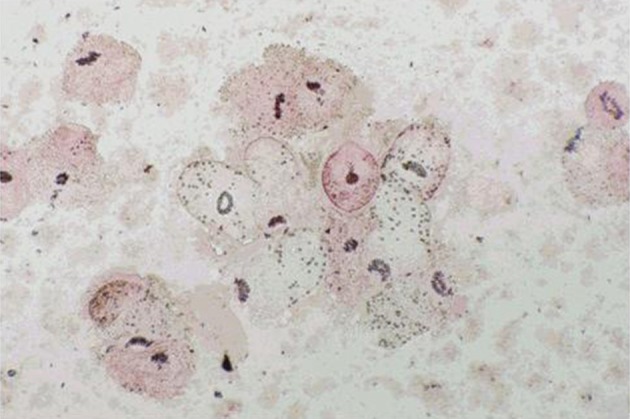
Protoscoleces After 18 h Exposure to Isolated Bacteria

**Figure 3.**
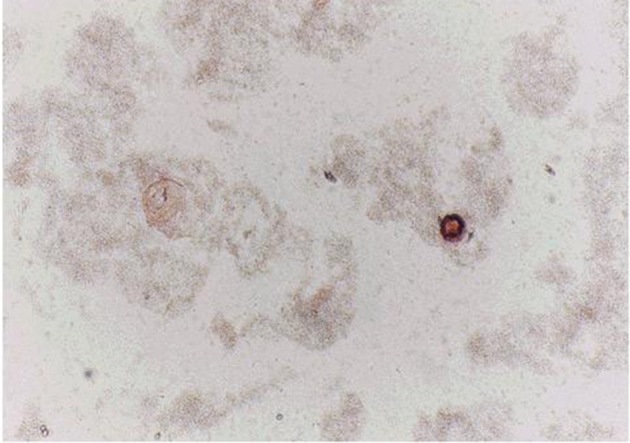
Protoscoleces After 30 h Exposure to Isolated Bacteria

**Figure 4.**
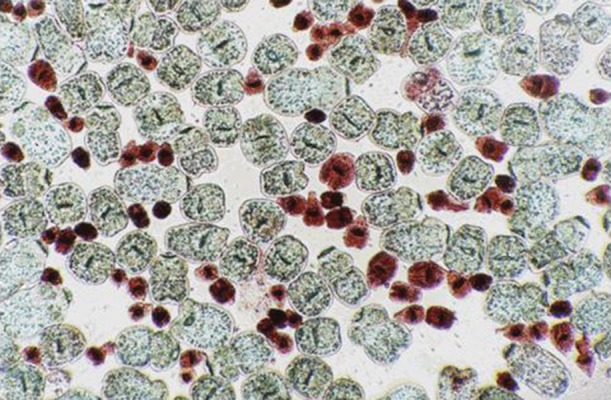
Protoscoleces Without Exposure to Isolated Bacteria

Protoscoleces incubated with all types of isolated bacteria totally degenerated, and the degeneration process began after six hours (Figures 1, 2 and 3). All protoscoleces in control groups remained intact and viable after 48 hours; however, 50% of the protoscoleces in control groups were intact and viable even after one week (Figure 4). The following bacteria degenerated the protoscoleces in shorter times than others: *E. coli*, *K. pneumonia*, *E. aerogenes*.

## 5. Discussion

The results of this study demonstrated that majority of hydatid cysts in the selected region are involved with bacterial infections. Although, infection of a hepatic hydatid cyst, especially in human cysts, is an uncommon complication, if the hydatid elements have not spontaneously ruptured into the biliary ductal system; but, this study showed that this condition is very common in animals, especially in this region. Sometimes, the cysts may heal spontaneously by inconspicuous rupture and evacuation, or degenerative or necrotic processes, probably caused by infection or immunological processes, leading to solidification or calcification. Infection of the cyst with bacterial flora from bile or bronchial tree is usually caused by the communicating rupture. The consequence of infection is degeneration of the cyst, leading to partial or total calcification of the lesion.

According to Marti-Bonmati, rupture of the cysts as a complication occurs in 50%-90% of cases, and infection develops only after rupture of both the pericyst and endocyst (5%-7% of cases) (8). Reports on the bacterial infection of humans’ hydatid cysts are not frequent. The records of 480 patients suffering from liver hydatid diseases in a university hospital of Zaragoza, Spain, were reviewed. Only 42 patients (less than 10%) fulfilled the intracystic bacterial infection criteria (13). In another report, 21 cases of hepatic abscesses were treated during a period of four years at the University of Patras, Greece, including 10 cases with abscesses caused by suppurated echinococcal cysts, corresponding to 21% of the total number of 47 cases of echinococcal cysts (14). The most reports of infected hydatid cyst in human are as case reports (15-17). Specially, records on the bacterial infections of animals hydatid cysts are very rare. For example, there are a few case reports from Turkey, India, Taiwan, etc. (9, 18-20). Types of the reported bacteria were *Yersinia* sp., *K. pneumonia*e, *E. coli*, *Salmonella typhi*, *Haemophilus influenza*, *Clostridium ramosum* and *S. milleri*.

In Iran, the mean prevalence of hydatidosis of sheep in different regions was 8.1%, varying from 1 to 27.5% (21-25). According to our results, almost 7% of the sheep, as the main intermediate host of *E. granulosus*, were infected with hydatid cysts. This rate was almost equal to other reports from Iran. Although, a previous study showed higher prevalence of hydatid cysts in Lorestan province (25.3%), the capital of which is Borujerd (22). However, prevalence of the disease in south of Iran was lower than western and northern parts and were reported about 2%-4% in different slaughtered livestock (23). In buffalos, 12.4% of the animals in the west and 11.9% in the north of Iran were infected with hydatid cysts (22, 24). However, in camels, the prevalence rate was reported 34.2% (26). Nevertheless, because of the low population of buffalos and camels and limited distribution of these animals in Iran, it seems that these intermediate hosts may not play important roles in the epidemiology of hydatidosis in Iran.

Fertility of cyst is an important factor affecting the stability of *Echinococcus* cycle in each region. Depending on the geographical situation, type of the infected host, and site, size and type of the cyst, fertility rates may differ. In Sardinia (Italy), the percentage of sheep with fertile cysts were reported only 10.25%, meanwhile, the purulent/caseous cysts were 12.7%, calcified cysts 59.7% and sterile cysts 28% (27). Kamhawi et al. reported that 38.1% of 3561 hydatid cysts recovered from 579 infected sheep were fertile, compared with only 4.5% of 155 cysts recovered from 38 infected goats, and 8.7% of 252 cysts recovered from 36 infected cattle (28). According to Dueger from Central Peruvian Andes, Peru, cyst fertility of the ovine cystic echinococcosis was 43.8% in hepatic origin and 56.2% in pulmonary origin cysts, indicating the importance of sheep as an intermediate host (29). Importance of the disease in southwest of Iran and the neighbor country, Iraq, was also considerable according to Rafiei et al. and Yacoub et al. reports in humans (30, 31).

On the other hand, mean prevalence of *E. granulosus* in the definitive hosts, domestic dogs, was about 23 % in Iran, varying from 3% to 50% depending on the local condition (30-33). For example, in some western parts of Iran, it was reported up to 48% (30). According to Dalimi et al. in a recent report, only 19% infection rate to *E. granulosus* was found in the mentioned region (32). Because of different distributions of domestic dogs as shepherd or guard dogs in houses, in different parts of the country, and traditional nomadic life or extended urbanization, interactions between dogs and humans are too variable (26). Therefore, hydatidosis/echinococcosis has been one of the most important zoonotic diseases, prevalent in different parts of Iran, including west (34, 35).

The use of protoscolecides is necessary in some conservative surgical procedures and in Puncture, Aspiration, Injection, Reaspiration (PAIR). Unfortunately, there has been no ideal protoscolecide agent with high effectiveness and safety to date. Degenerative effects of bacteria on protoscoleces could introduce a novel protoscolecide and increase the safety of surgeries. The mechanisms by which bacteria degenerates the protoscoleces is unknown. These mechanisms could include the effects of endotoxin or exotoxin, some enzymes, or other biochemical mediators on viable protoscox. Because the majority of isolated bacteria in this study were exotoxin producers, one strong probability was the degenerative effect of toxin on protoscoleces.

We have an ongoing research in which the main goal is extracting and fractionating the isolated bacteria by-products and challenging with viable protoscoleces. This work may explain which factors precisely cause degeneration of protoscoleces and sterilization of the fertile hydatid cysts. Extracting such material from the bacteria isolated from hydatid cysts and using them as protoscolecides, increases the hydatid surgery safety. These bacteria can even be used in biological control of parasites in the intermediate hosts.

This study indicated that high percentage of hydatid cysts in both provinces were bacteriologically infected. The common isolated bacteria were *E. coli* and *K. pneumoniae*. The bacteria could degenerate the protoscoleces *in vitro* during short-time incubation. Infection rates of the hydatid cysts were not statistically different in the two regions, Hamedan and Borujerd, two neighbor provinces.
